# Editorial: Computational intelligence for signal and image processing, volume II

**DOI:** 10.3389/fncom.2025.1581047

**Published:** 2025-03-11

**Authors:** Deepika Koundal, Jussi Tohka

**Affiliations:** ^1^School of Computer Science, UPES, Dehradun, India; ^2^A.I. Virtanen Institute for Molecular Sciences, University of Eastern Finland, Kuopio, Finland

**Keywords:** computational intelligence, signal processing, image processing, artificial intelligence, machine learning

## 1 Introduction

The second volume of this Research Topic features eight research articles that explore the use of Computational Intelligence in Signal and Image Processing II applications (Koundal and Ding, 2023; Wang et al., 2023). This edition explored brain-inspired algorithms and examined how they have driven the development of new methodologies in image/video and signal processing (Pan et al., 2024; Saikumar et al., 2022). It emphasized the significant potential of brain-inspired algorithms to transform multiple fields to drive innovation and enhance efficiency. Advancements in Artificial Intelligence (AI) and machine learning have significantly impacted on a variety of fields, ranging from healthcare and emotion recognition (Bing et al., 2024; Xia et al., 2023; Zhu, 2023) to image encryption (Chu et al., 2024) and human activity classification. By highlighting the interconnectedness of deep learning, neuro-fuzzy systems, neural networks, and other AI methods, it underscored their essential role in understanding and modeling the complexities of brain functions (Ye et al., 2024; Wen et al., 2024; Hao et al., 2023). This work connected neuroscience and technology by examining how brain insights can inspire the creation of novel algorithms and applications across diverse fields (Zhu, 2024; Hu et al., 2022). These innovations represent a significant leap forward in their respective domains, offering practical solutions with potential for real-world applications (Gezawa et al., 2023; Song et al., 2025).

## 2 Contributions

Cheng introduced a weighted closest neighbor algorithm to predict emotional distribution in abstract paintings. Emotional features have been extracted and assigned K-values that are followed by an encoder-decoder model that utilized a pre-trained network to enhance classification. Incorporating a blank attention mechanism, the model accurately identified emotional semantics- outperforming existing methods. This approach addressed the challenge of emotion recognition in abstract art. However, limitations are positional link detection and dataset constraints which suggest future expansion for more comprehensive classification. Sun et al. introduced a classification system for epileptic electroencephalography (EEG) signals using an attention network that has integrated nonlinear dynamic and time-frequency features. The system consisted of three modules: a parallel convolutional network for high-resolution Hilbert spectrum extraction, a residual-connected convolution module for nonlinear dynamic feature learning via grayscale recurrence plots, and a self-attention fusion module. The given system significantly improved the classification accuracy on multiple EEG databases that offered a promising approach to aid epilepsy diagnosis and treatment with broad clinical applications. Yaqoob et al. introduced an automated framework for Alzheimer's disease (AD) stage prediction using a Fuzzy Entropy-controlled Path-Finding Algorithm (FEcPFA) and ResNet-Self architecture. This method addressed dataset imbalance through data augmentation, incorporated a self-attention module to extract key information and Bayesian optimization (BO) to optimize hyperparameters. This framework improved the diagnosis accuracy, reduced computational time, and offered potential for early AD detection, though challenges like overfitting remain. Future improvements include using more diverse MRI datasets. Hou et al. developed an improved Dijkstra-based image encryption algorithm for color images that addressed the inefficiencies of traditional methods to treat color planes separately. Their approach integrated a new 1D chaotic system with enhanced randomness and an adaptive diffusion algorithm. The Dijkstra algorithm is used for cross-plane pixel scrambling to ensure better security and encryption efficiency. This method provided robust encryption for both medical images and standard RGB images by outperforming existing techniques in terms of security, quality, and robust to attacks especially in telemedicine applications. Roda and Geva introduced a pool-based semi-supervised active learning method for image classification using both labeled and unlabeled data. The approach involved clustering the latent space of a pre-trained convolutional autoencoder and applied a novel contrastive clustering loss (CCL) to enhance clustering even with limited labeled data. The system queries the most uncertain samples for annotation by iterating until the budget is exhausted. Empirical results show high accuracy with fewer labeled samples by offering an effective solution for image classification tasks with reduced annotation costs. Alarfaj et al. proposed a novel human activity recognition (HAR) approach using sensor-specific convolutional neural networks (CNNs) for accelerometers, gyroscopes, and barometers. Each CNN model is tailored to capture the unique features of its sensor type by addressing challenges with diverse data shapes. A late-fusion technique combined predictions from multiple models to significantly improving accuracy. Alsubai et al. introduced a facial emotion recognition system using a Modified ResNet model enhanced with quantum computing and advanced transfer learning. By integrating quantum convolutional layers with parameterized filters and employing residual connections, the system reduced the computation time and improved performance. The Modified up Sampled Bottle Neck Process (MuS-BNP) ensured computational efficiency. The model achieved superior accuracy, recall, precision and F1-score by overcoming challenges in distinguishing similar facial expressions. The results highlighted the system's potential for faster, more accurate facial emotion detection, using quantum computing and deep learning. Na et al. introduced FacialNet, a framework for facial emotion recognition (FER) using UNet image segmentation and transfer learning with EfficientNetB4. The approach is validated through cross-validation by offering high reliability and promised real-world applications in emotion-aware systems to enhance mental health assessments through more accurate emotion recognition. Zheng et al. introduced the Visual-Magnocellular Dynamics Dataset (VMD) with a multi-frame spike temporal encoding strategy to enhance dynamic visual information processing. They proposed the DT-MSTS backpropagation method for improved motion feature extraction in SNNs. Additionally, they integrated MG-SNN with YOLO to develop a retinal-inspired neural network for drone motion extraction and object detection. The study highlights the benefits of combining retinal mechanisms with SNNs, explores software-based deployment of neuromorphic chips, and suggests future directions for handling complex spatiotemporal data in real-world detection tasks.

## 3 Conclusion

This editorial presented nine research articles focused on the applications of Computational Intelligence for Signal and Image Processing. The studies highlighted significant advancements across various fields, from emotion recognition in abstract art to medical applications. Techniques such as deep learning, transfer learning, and quantum computing have shown great potential in improving accuracy, efficiency, and security. Despite their successes, challenges like dataset limitations, overfitting, and computational time remain. Future work includes expanding datasets, and refining models to enhance applicability in real-world settings across domains such as healthcare and mental health.
